# Modification of Sulfonated Polyethersulfone Membrane as a Selective Adsorbent for Co(II) Ions

**DOI:** 10.3390/polym13203569

**Published:** 2021-10-16

**Authors:** Gadeer R. Ashour, Mahmoud A. Hussein, Tariq R. Sobahi, Khalid A. Alamry, Sara A. Alqarni, Mohd Rafatullah

**Affiliations:** 1 Chemistry Department, Faculty of Science, King Abdulaziz University, Jeddah 21589, Saudi Arabia; gashour0001@stu.kau.edu.sa (G.R.A.); tsobahi@kau.edu.sa (T.R.S.); kaalamri@kau.edu.sa (K.A.A.); 2 Polymer Chemistry Lab., Chemistry Department, Faculty of Science, Assiut University, Assiut 71516, Egypt; 3 Department of Chemistry, College of Science, University of Jeddah, Jeddah 21959, Saudi Arabia; sasalqarni@uj.edu.sa; 4 School of Industrial Technology, Universiti Sains Malaysia, Penang 11800, Malaysia

**Keywords:** sulfonated polyethersulfone, chemical modification, amberlite-IRA-401, adsorption capacity, Co(II)

## Abstract

In the current study, a variety of sulfonated polyethersulfone (SPES)-based ion-exchange membranes were prepared and utilized as efficient and selective solid adsorbents for the detection of Co(II) ions in aquatic solutions. SPES membranes were treated with a variety of cations at a 2:1 ratio overnight. The produced materials were assessed via XRD, FT-IR, SEM, and TGA analyses. The structure of these materials was confirmed by FT-IR and XRD, which also confirmed the inclusion of Na^+^, NH_4_^+^, and amberlite on the SPES surface successfully. TGA analysis showed that the thermal stabilities of these materials were enhanced, and the order of stability was NH_4_-SPES > SPES > Na-SPES > A-SPES. Furthermore, the efficiency of these modified membranes for the determination and adsorption of a variety of metal ions was also examined by the ICP-OES analytical technique. A-SPES expressed a powerful efficiency of adsorption, and it showed an efficient as well as quantitative adsorption at pH = 6. Moreover, A-SPES displayed the highest adsorption capacity of 90.13 mg/g for Co(II) through the Langmuir adsorption isotherm.

## 1. Introduction

Polysulfone (PS) is a huge group of sulfur-containing polymers, which are aromatic resins with sulfone and ether links in the backbone. PS is divided into two categories: polyarylethersulfones (PAESs) and polyethersulfones (PESs) [1]. PES is one of the most studied and commonly utilized materials in the production of synthetic polymer membranes [2,3]. To use PES for membranes, it must be modified by adding different functional groups to the polymer, such as carboxylated (-COOH), hydroxylated (-OH), specially sulfonated (-SO_3_H), and aminated (-NH_2_) groups, or a combination of them [4]. Because of its low cost, ease of manufacture, predictable composition, outstanding mechanical strength, and strong chemical and thermal stabilities, sulfonated polyethersulfones (SPESs) have received a lot of research interest [5]. There are various methods to introduce sulfonic acid groups (SO_3_) into polysulfones, with one of them using sulfuric acid and chlorosulfonic acid at room temperature to produce SPES [6]. Then, SPES is used as a polymer composite for cation-exchange membranes (CEMs) [5,7,8]. In a previous study, a sulfonated polyethersulfone membrane was prepared for the dehydration of a water/ethanol mixture by pervaporation. SPES polymers were applied as an ion-exchange membrane and a pervaporation membrane. The addition of a sulfuric acid group into the polymer unit improved the polysulfone membrane hydrophilic property and made it a good material for dehydration [9]. Many studies have focused on improving polymer matrix properties by introducing a number of hydrophilic groups on SPES. The sulfonated group has an active hydrogen ion that can be replaced by lithium, potassium, sodium, ammonium, large-group polyurethane, etc. [3,10]. Moreover, it can cover the surface of SPES with several materials, such as PES [11].

Lithium chloride, potassium chloride, sodium chloride, lithium sulphate, potassium sulphate, and sodium sulphate are salts used to form cation-exchange membranes through their interaction with SPES. The co-ion and counterion influence on permselectivity in the cation-exchange membrane was investigated using these model materials and salts, and no new species effects were found in the salt investigations [12,13]. Polyarylethersulfones with sulfuric acid groups up to 1.2 per repeating unit were successfully prepared and substituted the hydrogen ion in SO_3_–H for the sodium ion in SO_3_–Na and showed higher water uptake and more thermal stability than those of the acid form when casting from solution. SPES with an ammonium group as a hydrophilic moiety achieves an improvement in the polymer matrix hydrophilic properties [10]. The combination of ion-exchange materials with other water treatment methods, such as ultra-filtration polyethersulfone membranes, has piqued the interest of scientists due to their increased operational capacity, longer equipment life, and reduced cost. As such, the researchers have used ion-exchange resins to remove various organic matters. For example, amberlite-IRA-401 is one of the most famous ion-exchange resins utilized in water treatment, but to date, no study has prepared amberlite with an SPES membrane [14]. Heavy metals are a category of contaminants that are of great concern because of their near-permanent persistence in the environment [15]. Industrial operations that discharge heavy metals into the ecosystem pose a major environmental concern. Over the last few decades, heavy metal ion determination and separation from aqueous solutions have been established through a variety of methods [16]. Metal ions in aqueous systems have been studied using a variety of analytical techniques, such as ion chromatography, including spectrophotometry, anodic stripping voltammetry, and inductively coupled plasma (ICP)–optical emission spectrometry (OES) [17,18,19,20]. Cobalt can be found in many situations, and it has a bad effect on human health; to prevent harmful consequences, analytical technology with high sensitivity and selectivity is needed [21,22]. As a result, detecting small quantities of cobalt traces in the environment is essential. To date, a variety of techniques based on various analytical methodologies have been used to identify Co(II) ions [23,24,25,26]. This study aims to produce a variety of cation-exchange membranes, an efficient and selective solid adsorbent for the determination and detection of Co(II) ions in aquatic solutions. The incorporation of sodium, ammonium, and amberlite into supramolecular materials is expected to result in interesting characteristics, such as enhanced TG and increased decomposition temperatures, which are investigated using TGA, as well as polymer morphology, which is observed using FE-SEM measurements. The characteristics of the materials are compared with the sulfonated polyethersulfone membrane prepared via the direct sulfonation route.

## 2. Experiment

### 2.1. Materials

Polyethersulfone (PES, Veradal 3000 MP) with MW 66 kD was supplied by Solvay Chemicals Limited, Asia Pacific Pte. Ltd., SINGAPORE, SINGAPORE, and employed with no additional treatment. Chlorosulfonic acid (CSA, Aldrich, New York, NY, USA) was used as a sulfonating agent, sulfuric acid (98%). The cation-exchange materials were prepared using a precipitating agent (deionized water). For the material synthesis, chloroform and dichloromethane were employed as solvents. Methanol was used to wash the polymer. Ammonium hydroxide and sodium chloride were used for the ion-exchange process. All the solutions were prepared in doubly distilled water using Pyrex glass apparatus. Amberlite-IRA-401 ion-exchange resin was obtained (CSA, Aldrich, New York, NY, USA). Stock Standard solutions, namely Cd(II), Ni(II), Fe(III), Cr(III), Cu(II), Co(II), Pb(III), Zn(II), and Al(III) (1000 mg L^−1^ of each), were supplied by Sigma-Aldrich (WI, Milwaukee, WI, USA) and were employed without any treatments. Other reagents were utilized as typically produced without additional purity.

### 2.2. Chemical Modifications of PES

#### 2.2.1. Preparation of the Sulfonated PES (SPES)

The SPES was prepared using the normal sulfonation process of dissolving PES in concentrated H_2_SO_4_ and adding chlorosulfonic acid ClSO_3_H, sulfonating agents, with constant stirring for 20 h at room temperature (25 °C). After that, the final sample was realized in ice deionized water [5] and totally washed using distilled water several times until a pH of ~6–7 and a fixed weight was reached. SPES samples were kept in a vacuum oven for drying. A variety of factors can affect the degree of sulfonation (DS) of the modified PES, including polymer concentration, the used solvent, the time of the reaction, the temperature of the reaction, and the sulfonating agent strength. The method by Unnikrishnan et al. was followed to obtain our SPES. Hence, the expected DS was obtained with a maximum of 60% [5]. Furthermore, the thickness of the obtained membranes was found to be 170 μm.

#### 2.2.2. Preparation of PES Containing Pendant Sodium Sulfonate Group (Na-SPES)

Prior to the preparation of the selected proton ion-exchange polyethersulfone, the SPES was washed using methanol and dried for 5 min. The SPES was dissolved in a small amount of chloroform. After the material was dissolved in the solvent, the precipitation process occurred in the presence of 0.1 M sodium chloride solution for 24 h. The final product was further dried for 24 h [10].

#### 2.2.3. Preparation of PES Containing Pendant Ammonium Sulfonate Group (NH_4_-SPES)

Prior to the preparation of the selected proton ion-exchange polyethersulfone, the SPES was washed using methanol and dried for 5 min. The SPES was dissolved in a small amount of chloroform. After the material dissolved in the solvent, the precipitation process occurred in the presence of 0.1 M ammonium hydroxide solution for 24 h. The final product was further dried for 24 h [10].

#### 2.2.4. Preparation of SPES Containing Pendant Amberlite-IRA-401(A-SPES)

After drying, the SPES was additionally washed using methanol for a further 5 min. After that, the SPES was dissolved in a small amount of DCM. Then, the amberlite (0.1 M) was added to the polymer solution with heat for one hour. A modified SPES membrane was prepared through the coating. The final product was additionally dried for 24 h.

### 2.3. Adsorption Method Procedure

Standard sample solutions (Cr(III), Al(III), Co(II), Cd(II), Pb(III), Fe(III), Cu(II), Zn(II), and Ni(II)) were produced in deionized water (18.2-million-ohm cm (M cm)—the resistivity of the deionized water) and stored in the dark at 4 °C. Then, 5 mg/L of the Co(II) (or another metal ion) standard solution was produced and individually combined with 30 mg of all tested material phases for the selectivity investigation. Moreover, 5 mg/L of the Co(II) ion standard solution was produced and calibrated with a suitable buffer solution to a pH value ranging from 3.0 to 8.0. Each standard solution was prepared separately with 30 mg of an A-SPES derivative with an adsorption toward Co(II). At ambient temperature, all mixes were manually shaken for 1 h at 170 rpm. For the investigation of Co(II) adsorption capability under component design, 3, 5, 10, 15, 20, 25, 30, 50, 75, 100, 125, 150, and 175 mg/L of Co(II) were used as standard solutions and produced as described before, with a regulated pH of 6.0, and separately combined with 30 mg of A-SPES material.

### 2.4. Instrumentation

Fourier transform infrared (FT-IR: PerkinElmer, Spectrum 100, New York, NY, USA) spectrum was obtained in the wave number of 4000–400 cm^−1^. The thermal stability tests of the SPES and ion-exchange membranes were carried out using DTG-60H, Germany. Samples were placed in an alumina macro pan and heated from 25 °C to 800 °C at a ramp rate of 10 °C/min in a nitrogen atmosphere. The morphology of the entire polymer matrix was analyzed by field-emission scanning electron microscopy (FESEM) (Jeol JSM-7600F, Tokyo, Japan). For the XRD powder determination, the data were obtained using an X-ray diffractometer (Mannai Technical Services; model type RIGAKU ULTIMA_IV, New York, NY, USA) with Ni-filtered CuKα radiation in a range (2*θ*) between 5° and 80°. The detection of the metal ions was conducted using an inductively coupled plasma–optical emission spectrometer (ICP-OES), Optima 4100 DV, Perkin Elmer, New York, NY, USA.

## 3. Results and Discussion

### 3.1. Chemical Modification and Characterization

**Figure 1:**
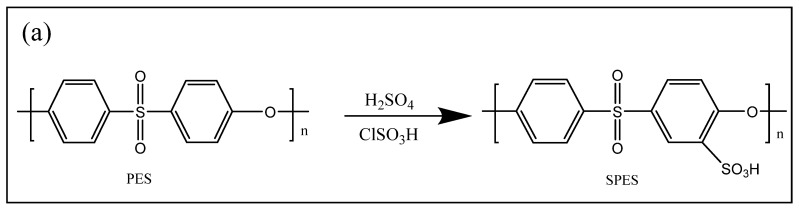

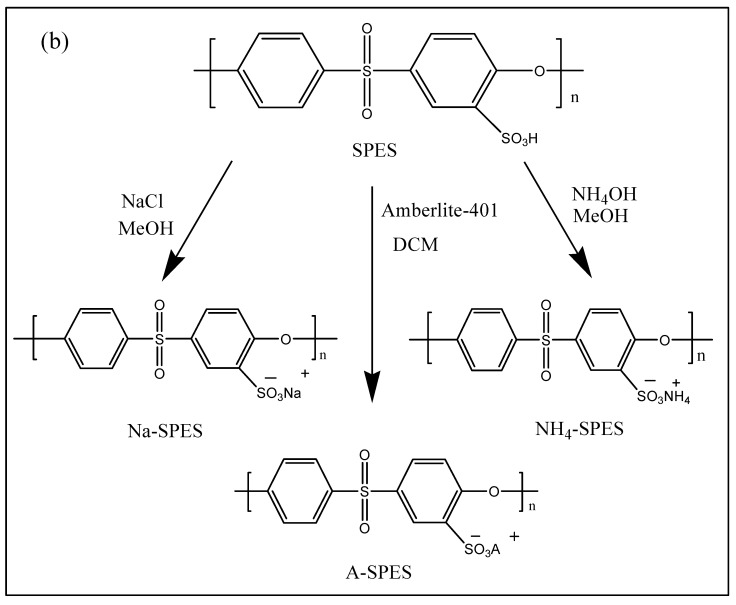
(**a**) Preparation of SPES. (**b**) Preparation of modified SPES membranes (Na-SPES, NH_4_-SPES, and A-SPES).

In this study, the sulfonation process of pure PES was carried out in the presence of concentrated sulfuric acid as well as chlorosulfonic acid at room temperature; herein, the sulfonated PES membrane is referred to as SPES, and it was synthesized successfully. The main purpose for such an important PES chemical treatment is to create a modified PES polymer surface with a negative charge as previously reported in the literature [5,27]. A variety of factors affect the DS of the modified PES, including polymer concentration, the used solvent, the time of the reaction, the temperature of the reaction, and the sulfonating agent strength. The method by Unnikrishnan et al. was followed to obtain our SPES. Hence, the expected DS was obtained with a maximum of 60% [5,27]. The chemical structures for both PES and its modified SPES are illustrated in Figure 1a.

In membrane environmental treatments, ion-exchange membranes have long been employed in electrodialysis. Ion-exchange membranes used in desalination must have high selectivity and conductivity, excellent mechanical and chemical stability, and resistance to organic fouling in order to be effective. The selection of polymeric matrices, the modification of membranes with different functional groups, and the membrane surface layer were used as strategic instruments to accomplish these characteristics. Therefore, further modification was carried out for the SPES through cation-exchange methodologies. The interaction was displayed via the incorporation of sodium chloride, ammonium hydroxide, and amberlite-IRA-401 into the SPES to produce the desired modified PES containing the pendant sodium sulfonate group, ammonium sulfonate group, and pendant amberlite-IRA-401, respectively. Herein, these products are referred to as Na-SPES, pendant NH_4_-SPES, and A-SPES. Figure 1b shows the chemical equations for the desired chemical reactions.

**Figure 2:**
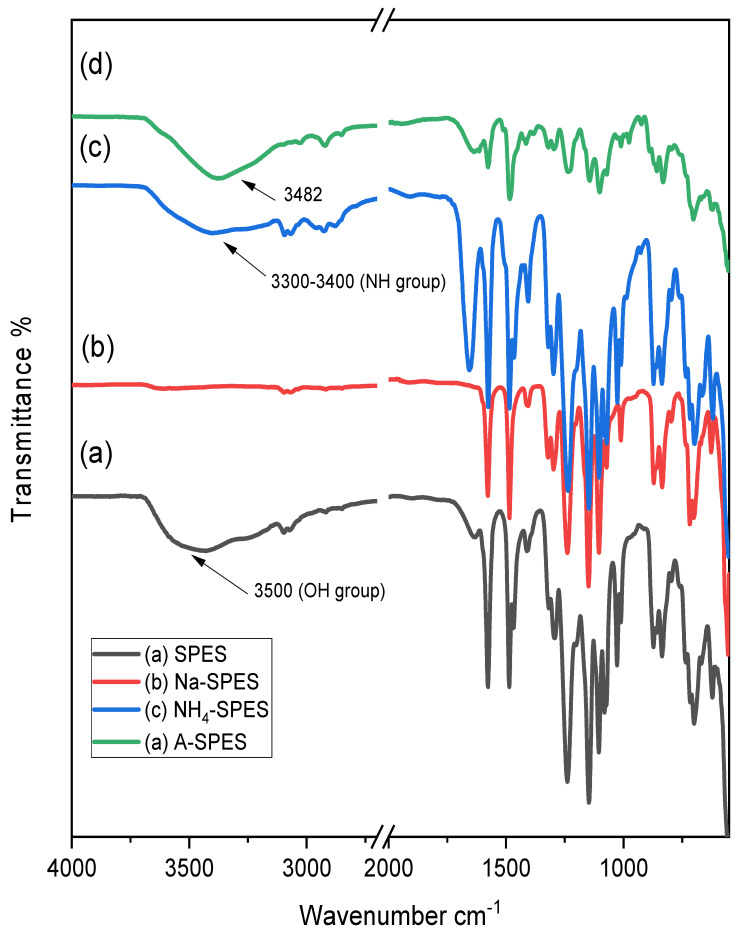
The spectra of FT-IR for (**a**): pure SPES, (**b**): NH_4_-SPES, (**c**): A-SPES, and (**d**): Na-SPES.

The resultant modified structures were investigated using the FT-IR technique. As presented in Figure 2, the two very broad ranges of 1570–1590 cm^−1^ indicate the symmetrical stretching of [C=C] from the aromatic skeleton of Na-SPES and NH_4_-SPES, respectively, whereas that around 1238 and 1232 cm^−1^ can be typically attributed to aryl oxide for Na-SPES and NH_4_-SPES, respectively. Strong absorption appearing at around 1148–1104 and 1145–1103 cm^−1^ was assigned to the stretching vibration of the aromatic sulfone (O=S=O) groups in the spectra of Na-SPES and NH_4_-SPES, respectively. However, the characteristic peaks of NH_4_-SPES of aromatic SO_3_^-^ stretching vibrations observed at 1070–1010 and 1071–1011 cm^−1^ were assigned to the stretching vibration of the sulfonate groups in the spectrum of Na-SPES and NH_4_-SPES, respectively, while the ether conformational peaks appear at around 1295–1318 cm^−1^ for both [5,8]. Regarding the NH_4_-SPES spectrum, the band at 1638 cm^−1^ mainly refers to the deformation of asymmetric -NH_4_^+^. Peaks in the range from 2800 to 3000 cm^−1^ provide extra evidence for the existence of NH_3_^+^ in the desired membrane. The strong and wide absorption band above 3500 cm^−1^ indicates the stretching of the SO_3_H hydroxyl group. Similarly, the ammonium-substituted SPES spectrum displays a strong absorption peak at 3300–3400 cm^−1^; this could be ascribed to N-H stretching, which was absent in Na-SPES. These results are in accordance with the literature [10]. A-SPES has different adsorption peaks that can be observed at 3482–1671 and 1410–2920 cm^−1^. The band at 3482 cm^−1^ indicates the hydroxyl -OH stretching vibration from the water, and the band at 1671 cm^−1^ indicates the OH bending vibration connected with SPES. In contrast, the bands at 1410 and 2920 cm^−1^ specify the stretching vibrations of C-H bonds [28,29]. All of the remaining peaks that are the same as those of SPES are due to the introduction of amberlite-IRA-401 to SPES. All the specific observations in the FT-IR results prove that SPES was synthesized successfully without any side reactions, and it was well incorporated into Na-SPES, NH_4_-SPES, and A-SPES membranes.

**Figure 3:**
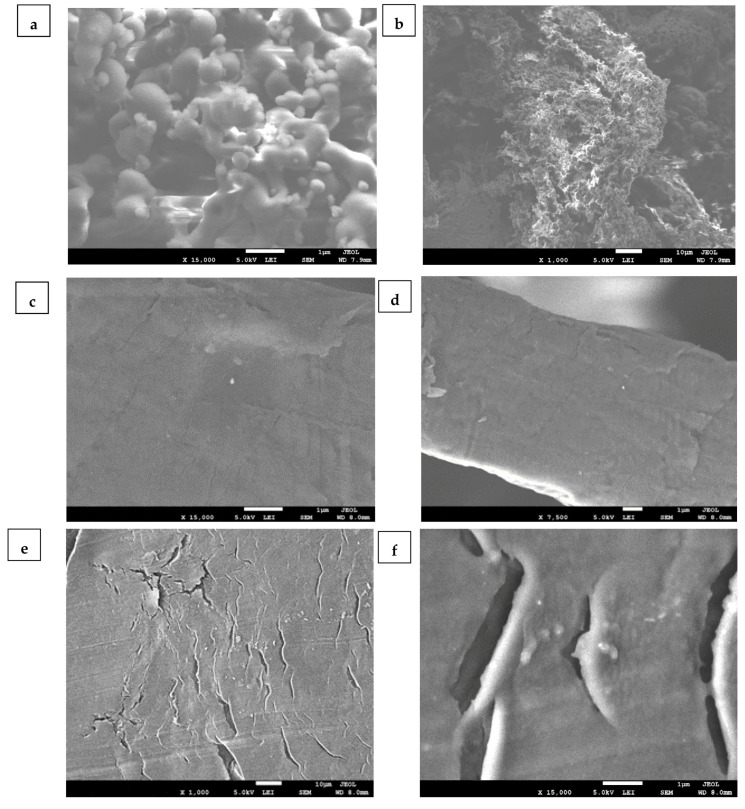

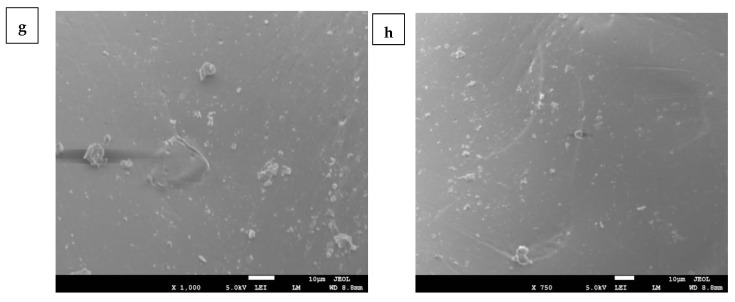
(**a**,**b**): Photos of SPES pore body, (**c**,**d**): images of Na-SPES, (**e**,**f**): images of NH_4_-SPES, and (**g**,**h**): images of A-SPES smooth body.

The SEM photos for the SPES surface show that the pores are distributed throughout the membrane after sulfonation, whereas the Na-SPES, NH_4_-SPES, and A-SPES membranes exhibit more uniform and homogeneous surfaces [10]. Thus, the pictures demonstrate how the SPES content affects the porous structure. Based on the images observed, Figure 2 also shows that the surface changed from rough to smooth with the incorporation of Na^+^, NH_4_^+^, and A into SPES. It is important to mention that the modification of SPES into sodium, ammonium, and amberlite SPES plays an essential role in membrane formation. The development of cracks indicates that polymer chains become entangled during membrane construction. Ion-crosslinking between polyethersulfones, when Na^+^, NH4^+^, and A are high enough to generate inter-polymer chain semi-crosslinking, is one probable source of polymer entanglement. Unless polymer entanglement occurs prior to membrane unification, a nodule forms on the non-crosslinked polymer, followed by a smooth cluster on the surface (see Figure 3).

These results show the successful formation of SPES, and the interaction between Na^+^, NH_4_^+^, and A with SPES was observed. Moreover, the change in the morphologies can be attributed to the increase in the hydrophilicity of the membrane polymers.

**Figure 4:**
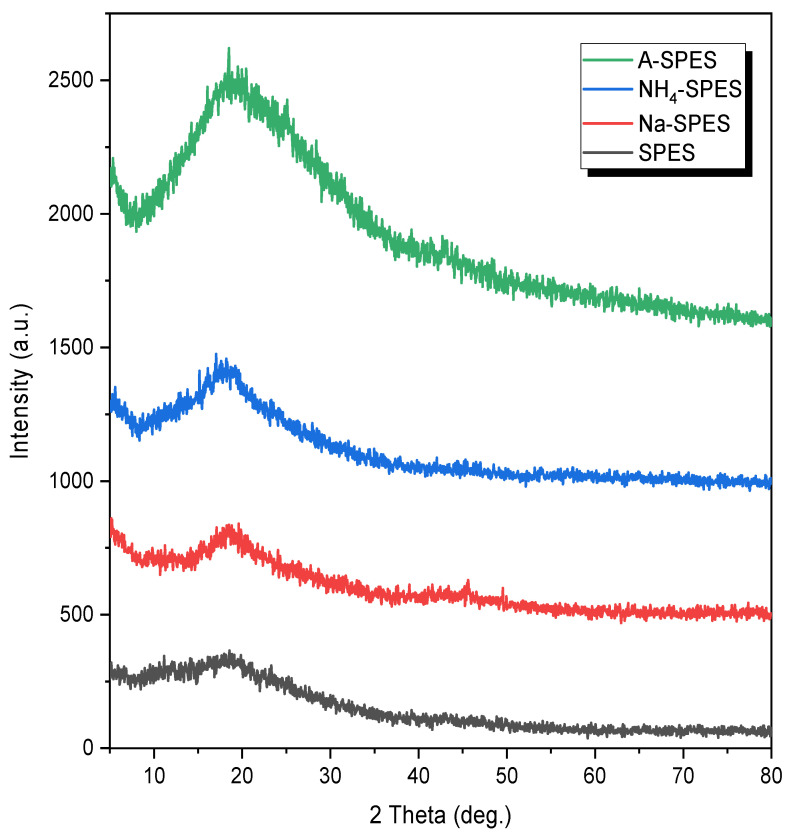
X-ray diffraction patterns of the modified PES materials.

The XRD analysis for the plain SPES versus Na-SPES, NH_4_-SPES, and A-SPES is illustrated in Figure 4.

The SPES has a broad halo peak in the range between 12 and 27°, which is attributed to the amorphous structure of SPES as a result of the existence of benzene rings as previously reported [3]. However, the NH_4_-SPES spectrum shows a sharp peak with a shift to 19°. Two peaks appeared after incorporating the SPES with Na^+^ and NH_4_^+^ at 2*θ* = 19°, and 46° indicates the existence of Na^+^ and NH_4_^+,^ and their incorporation with SPES. In the amberlite SPES spectrum, the substitution produced an amorphous structure that somewhat changed to a narrower peak at 20°, and 44° indicates the presence of amberlite and its interaction with SPES.

**Figure 5:**
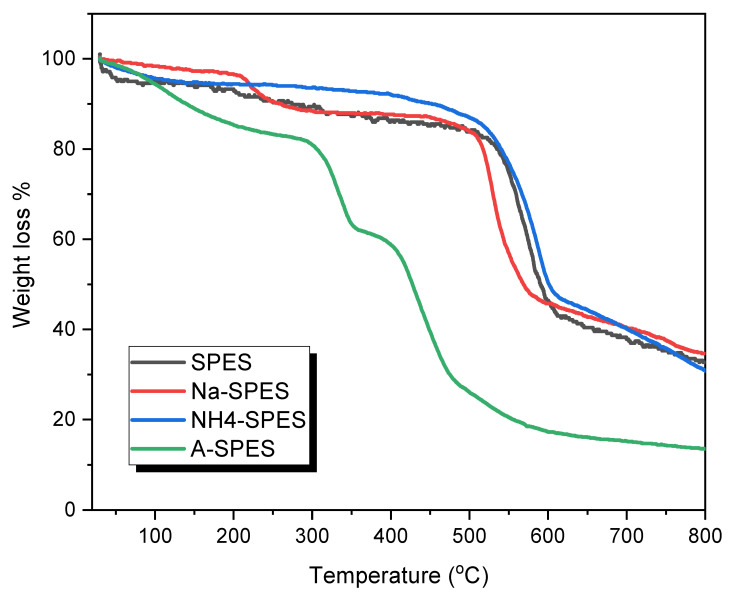
TGA thermograms of the modified PES materials.

TGA analysis was performed to assess the degree of alteration and to investigate the thermal stability of the ion-exchange membranes that were produced. The thermal stability test of the ion-exchange membranes was carried out starting from a normal temperature to 800 °C. The heating conditions were selected as 10 °C min^−1^ heating rate under a steady nitrogen atmosphere (Figure 5). In addition, Table 1 displays the temperatures (°C) for various percentages of decompositions from 10 to 50%, which are attributed to the weight losses from 10 to 50% degradation, respectively [30,31]. As observed in the figure, the TGA curves of SPES, Na-SPES, NH_4_-SPES, and A-SPES display a slight weight loss in the range of 3–5%, which almost stops before 160 °C as a result of the solvation process and/or the extraction of the attached adsorbed water. The temperatures for 5% weight loss represent the polymers’ decomposition temperatures (PDTs) [31,32].

The TG curves of SPES and NH_4_-SPES exhibited two-step degradations in the range of 400–550 °C, which indicated the degradation of the SO_3_H and SO_3_-NH_4_ groups of the membrane, and around 600 °C, which was due to the degradation of the residue main chain. In contrast, the TG curve of Na-SPES and A-SPES exhibited four-step degradations, with the first degradation at 200 and 290 °C, the second degradation at 250 and 354 °C, the third degradation at 500 and 400 °C, and the final degradation at 580 and 480 °C. For all ion-exchange membrane polymers, the typical decomposition rate for the first step is significantly quicker than that of the second step. Additionally, the high decomposition temperature indicates that A-SPES has the lowest thermal stability amongst all of the products. The decomposition becomes extremely high at around 650 °C and stops at about 750 °C, which is displayed by the thermal degradation of the main skeleton of the material. The results given in Table 1 highlight the temperature values for a variety of weight loss percentages (from 10 to 50%), and they indicate that the thermal stabilities of these products can be arranged in the following order: NH_4_-SPES > SPES > Na-SPES > A-SPES. Thus, the high decomposition temperature indicates that the A-SPES membrane has the lowest thermal stability in the region of 10–50%.

### 3.2. Surface Selectivity Study

Chemical modifications of polymeric materials through chemical reactions and/or the formation of polymer nanocomposite materials have been utilized across a huge number of industrial and environmental applications, with special attention paid to heavy metal determination from environmental sources. In the last few decades, solid-phase adsorbents based on these types of hybrid material structures have also been widely utilized to detect a variety of metal ions in aqueous solutions [33,34,35,36,37]. Based on the above-mentioned information, the SPES membranes (SPES, Na-SPES, NH_4_-SPES and A-SPES) were utilized as selective modified surface adsorbents to determine harmful Co(II) metal in aquatic solutions. As compared to the pure SPES, all produced SPES membranes had a considerable adsorption efficiency. The A-SPES membrane in particular had the highest adsorption effectiveness of all the membranes (SPES, Na-SPES, and NH_4_-SPES); consequently, as shown in Table 2, it was selected as the research model for modified surface selectivity in this current research paper.

**Figure 6:**
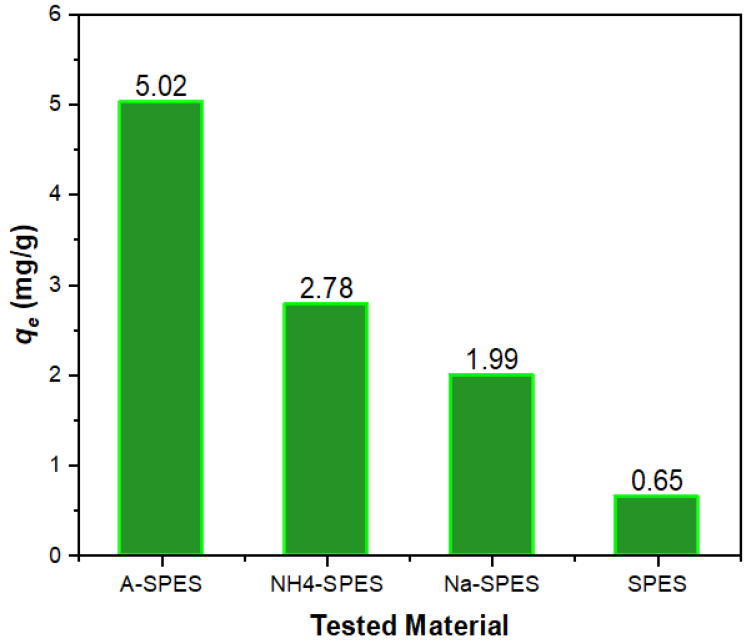
Surface selectivity study of SPES, Na-SPES, NH_4_-SPES, and A-SPES toward Co(II) ions.

The A-SPES derivative’s distribution coefficient selectivity toward various metal ions was determined. The following equation was used to determine the distribution coefficient (*Kd*):

(1) $$K_{d}=\frac{\left(Co-Ce\right)\;}{Ce}\times \frac{V}{M}$$

where *C_o_* and *C_e_* display the starting and definitive concentrations before and after filtration with the used material, respectively; the volume is presented as *V* (mL); and the adsorbent weight is presented as *M* (in g). Table 2 displays the values of the distribution coefficients for all of the metal ions involved in this research. As a result of the selectivity of the SPES membranes against Co(II), A-SPES was shown to be the most selective material against Co(II) ions among the SPES membranes evaluated. For the studied products, the order of increasing Co(II) ions is as follows: A-SPES > NH4-SPES > Na-SPES > SPES. Figure 6 depicts this arrangement.

**Figure 7:**
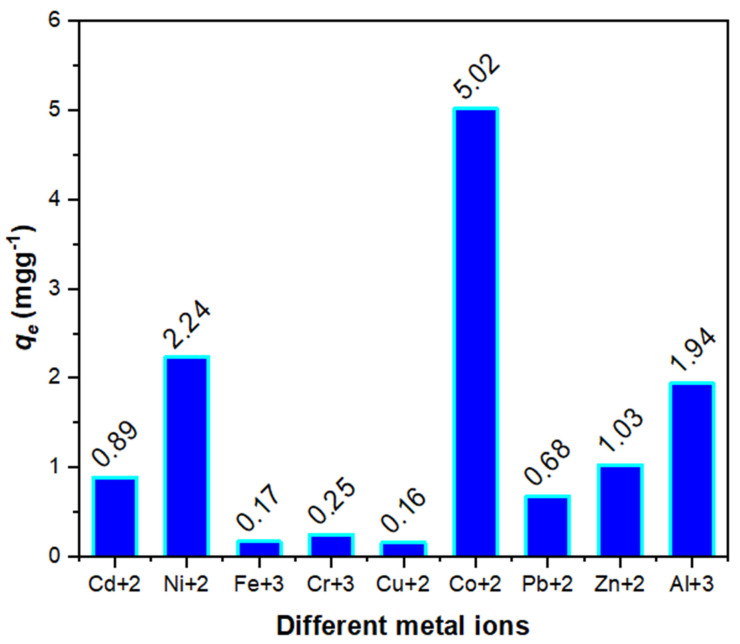
Surface study of selectivity for A-SPES against different metal ions.

A-SPES was also tested against the remaining nine ions, as shown in Table 2. Through the examined metal ions, A-SPES displayed the highest value for the distribution coefficient (51,489.60 mL/g) for Co(II). Furthermore, these findings suggest that when emulated to the overall studied metal ions in this work, the selectivity of this synthetic derivative toward Co(II) is the best amongst the other selected metal ions as illustrated in Figure 7.

#### 3.2.1. Effect of pH

**Figure 8:**
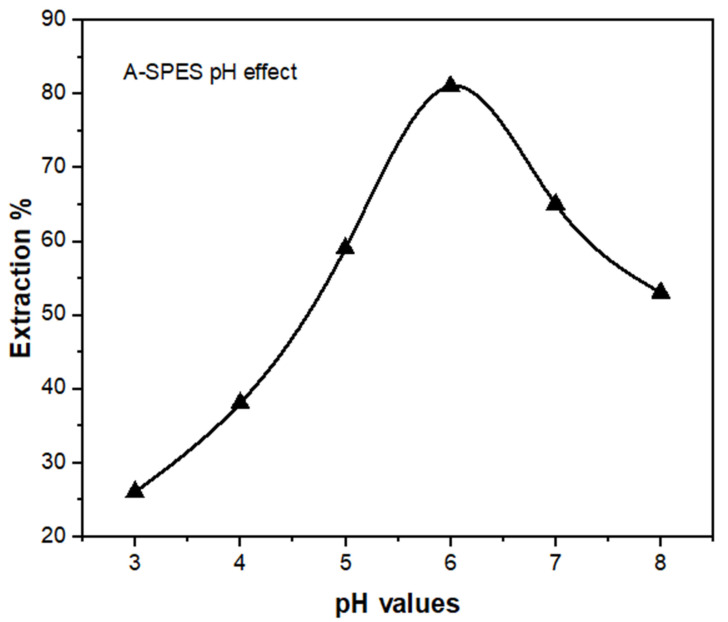
The effect of pH values on the adsorption of Co(II) ions (2 mg/L) on 30 mg of A-SPES at 25 °C.

The presence of H+ ions in a solution influences the adsorbed species and ionization degree, as well as metal ion detection in an aquatic environment by adsorption [38]. The impact of pH on Co(II) adsorption via the newly synthesized A-SPES compound was studied as a result. The impact of pH was investigated by altering the pH solution limit from 3.0 to 8.0 with matching buffer solutions, using a Co(II) concentration of 5 mg/L. Each standard solution was prepared separately with 30 mg of A-SPES. The influence of pH on the percentage of extraction is shown in Figure 8; pH has an immediate impact on the process of removal. The tests were carried out with 30 mg of A-SPES at 25 °C and the pH limit in the range from 3.0 to 8.0. With a rising pH value, the extraction percent of Co(II) notably rises, followed by a drop, as seen in Figure 6. The largest percentage of extraction of Co(II) (81%) occurs at pH 6.0, indicating that the A-SPES compound represents the highest selectivity for Co(II) at this pH level.

The possible protonated sites of electrostatic interaction, which are present on the sulfone groups of A-SPES at pH 6.0, and the species with negative charges (CoCl_4_^−^, the major structure for the Co(II) ions in HCl solution) explain the Co(II) extraction maximum percentage and electricity with A-SPES at this pH value. As a result, Co(II) is selectively eliminated from the matrix. Regarding the above-mentioned results, the pH value of 6.0 was investigated as the optimum for studying the remaining parameters that determine the maximal uptake on the A-SPES compound during standard operations.

#### 3.2.2. Determination of Adsorption Capacity

**Figure 9:**
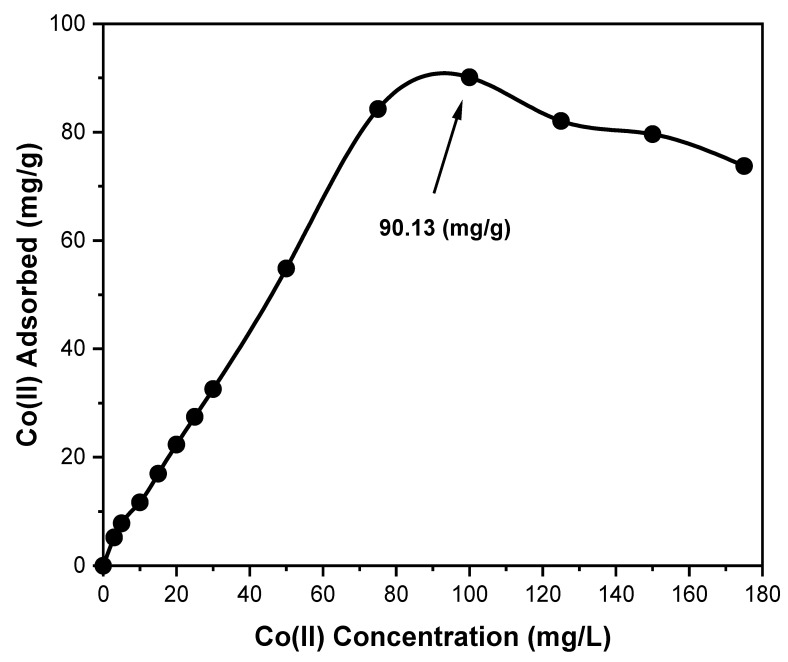
The Co(II) ion adsorption profile on 30 mg of A-SPES in relation to the concentration at 25 °C and pH 6.0.

Different quantities of Co(II) are separately combined with 30 mg of an A-SPES derivative at pH 6.0 in a batch process to evaluate the absorption capability of Co(II). Per the adsorption isotherm research, A-SPES has an adsorption capacity of 90.13 mg/g for Co(II), as displayed in Figure 9. This figure further shows that the A-SPES derivative’s Co(II) uptake capability decreases somewhat after overloading. The binding center fullness of A-SPES with the CoCl_4_^−^ species, especially at the maximum concentration of Co(II), 175 mg/L, explains this finding. After following this strategy, there may be a negligible effect of concentration on the maximal absorption capacity of the A-SPES derivative for Co(II). The A-SPES derivative’s stability was tested for more than three cycles, providing a roughly identical capacity, which indicates its high stabilization for complete additional use.

#### 3.2.3. Adsorption Isotherm Model

**Figure 10:**
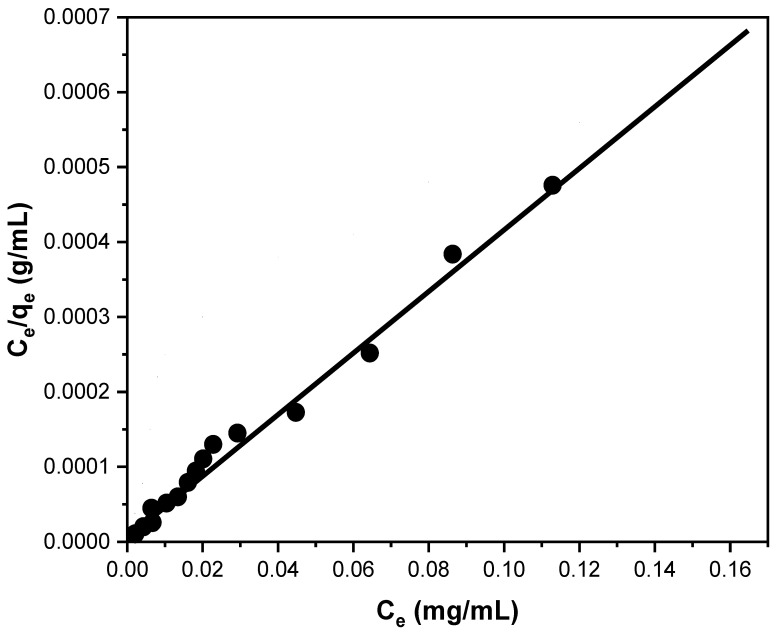
Isotherm model adsorption for Co(II) ion adsorption on 30 mg of A-SPES at 25 °C and pH 6.0. A variety of concentrations (0–175 mg/L) of Co(II) were used at static operations.

The study of isotherm models is crucial for anticipating outcome analysis. The Langmuir adsorption model can be used to characterize isotherms [39,40]. The Langmuir equation fits well the scientific results, as seen in Figure 10.

The Langmuir isotherm model determines how homogeneous non-interacting surface area locations are. The standard Langmuir adsorption isotherm is [41]

(2) Ce/qe=Ce/Qo+1/Qob

where *C_e_* represents the concentration of the metal ion in the solution at equilibrium (mg/mL), and *q_e_* represents the metal ion amount per gram for the adsorbent at equilibrium (mg/g). The Langmuir constants for A-SPES are represented by the symbol *Q_o_*, and they are also referred to as the Co(II) adsorption capacity maximum in mg/g, while the affinity parameter (l mg^−1^) is represented by the symbol *b*. Constants of the Langmuir A linear plot of *C_e_/q_e_* versus Ce with a slope and intercept equal to 1/*Q_o_* and 1/*Q_o_b*, respectively, may be used to compute *Q_o_* and *b*. Furthermore, the Langmuir adsorption isotherm model’s key features may be expressed in expressions of a constant separation dimensionless operator or equilibrium parameter, *R_L_*, which is defined as follows:

(3) $$R_{L}=1/\left(1+bCo\right)$$

where *b* is the constant of Langmuir, which depicts the nature of adsorption and different shapes of the isotherm, and *C_o_* is the concentration of the Co(II) ions at the initial stage. The origin of the adsorption isotherm is indicated by the value of *R_L_*, with values between 0 and *R_L_* in the form of 0 < *R_L_* < indicating preferential adsorption [42]. As illustrated in Figure 9, the least squares fit produces a linear plot from the equation of Langmuir, confirming the correctness of the Langmuir adsorption isotherm model for the adsorption operation. According to the aforementioned findings, mostly during the adsorption mechanism, there is a homogenous monolayer surface for the A-SPES product. The constants of Langmuir for *Q_o_* and *b* are 89.46 mg/g and 0.22 L/mg, respectively. For the adsorption of Co(II) on A-SPES, the correlation coefficient (R^2^) determined using the Langmuir model is 0.987, suggesting that the data may be matched with the Langmuir model. The value of *R_L_* for Co(II) adsorption on A-SPES is 0.05, indicating that the Langmuir model predicts a very advantageous adsorption operation. Furthermore, the Langmuir equation-calculated Co(II) adsorption capacity (92.63 mg/g) is in satisfactory correlation with the experimentally determined Co(II) adsorption capacity (90.13 mg/g) from the isotherm of the adsorption research. A comparison of A-SPES with previously reported adsorbents for Co(II) adsorption is presented in Table 3.

## 4. Conclusions

Sodium-, ammonium-, and amberlite-containing cation-exchange membranes were produced successfully based on sulfonated PES for the detection and removal of Co(II) ions. The morphological studies of the SPES, Na-SPES, NH_4_-SPES, and A-SPES cation-exchange membranes indicated that the integration of these positively charged species to the SPES surface led to a covering of the pores in the SPES body. In the XRD examinations, the crystallinity of Na-SPES, NH_4_-SPES, and A-SPES changed to a sharp peak. These modified materials are new, efficient materials for the determination of Co(II) ions in aquatic solutions. Furthermore, the ICP-OES instrument was investigated as an efficient way to determine the efficiency of such SPES membranes toward different metal ions. All of the fabricated modified materials showed higher selectivity toward Co(II) ions compared to the SPES. At pH 6, the A-SPES membrane had an extremely high efficiency of adsorption, with most of the amount adsorbed. A-SPES had an adsorption capacity of 90.13 mg/g for Co(II), and its isotherm of adsorption was in agreement with the Langmuir adsorption isotherm.

## Figures and Tables

**Table 1:** Thermal properties of modified SPES membranes.

Material	Temperature (°C) for Various % Decompositions *				
	10%	20%	30%	40%	50%
SPES	258	535	560	542	592
Na-SPES	256	517	529	575	569
NH_4_-SPES	445	540	566	584	601
A-SPES	136	304	335	390	426

* Determined from the TGA curves.

**Table 2:** Study of the selectivity on the adsorption of SPES, Na-SPES, NH_4_-SPES, and A-SPES against different metal ions at 25 °C (N = 3).

Metalions	*q_e_* (mg/g)				*K_d_* (mL/g)			
	A-SPES	NH_4_-SPES	Na-SPES	SPES	A-SPES	NH_4_-SPES	Na-SPES	SPES
Cd^+2^	0.89	-	-	-	368.31	-	-	-
Ni^+2^	2.24	-	-	-	447.68	-	-	-
Fe^+3^	0.17	-	-	-	52.82	-	-	-
Cr^+3^	0.25	-	-	-	128.35	-	-	-
Cu^+2^	0.16	-	-	-	51.74	-	-	-
Co^+2^	5.02	2.78	1.99	0.65	51,489.60	566.09	218.43	268.87
Pb^+2^	0.68	-	-	-	303.09	-	-	-
Zn^+2^	1.03	-	-	-	155.36	-	-	-
Al^+3^	1.94	-	-	-	202.45	-	-	-

N, number of reading.

**Table 3:** Selected adsorption capacity reports for Co(II) with different adsorbents.

Adsorbents	Adsorption Capacity (mg/g)	References
Magnetic multiwalled carbon nanotube/iron oxide composites	10.61	[43]
Polyvinyl alcohol/chitosan magnetic composite	14.39	[44]
Magnetic cyanoethyl chitosan beads	17.92	[45]
Xanthate-modified magnetic chitosan	18.50	[46]
Natural vermiculite	49.50	[47]
Magnesium hydroxide powders	125.0	[48]
A-SPES	89.46	This study

## Data Availability

The data presented in this study are available on request from the corresponding author.
